# Supplementary material for: Chromatin network retards nucleoli coalescence
Source: Nat Commun. 2021 Nov 24;12:6824. doi: 10.1038/s41467-021-27123-9 (PMC8613291; doi:10.1038/s41467-021-27123-9)
Supplement: Supplementary file 1 — Supporting Information [file 41467_2021_27123_MOESM1_ESM.pdf]

Supplementary Material for:
Chromatin Network Retards Droplet Coalescence

Yifeng Qi and Bin Zhang*

Departments of Chemistry, Massachusetts Institute of Technology, Cambridge, MA 02139, USA

(Dated: October 17, 2021)

* binz@mit.edu

The Diploid Human Genome Model Parameterized with Hi-C Data

The energy function of the genome model is defined as

$$U_{\text{Genome}}(\mathbf{r}) = U(\mathbf{r}) + U_{\text{intra}}(\mathbf{r}) + U_{\text{inter}}(\mathbf{r}) + U_{\text{Xi}}(\mathbf{r}) + U^{\text{specific}}_{\text{inter}}(\mathbf{r}). \tag{S1}$$

$U(\mathbf{r})$ represents a generic potential applied to each chromosome to ensure the polymeric topology of chromosomes:

$$U(\mathbf{r}) = \sum_i \left[u_{\text{bond}}(r_{i,i+1}) + u_{\text{angle}}(\vec{r}_{i,i+1}, \vec{r}_{i+1,i+2}) + u_c(r_i)\right] + \sum_{j>i} u_{\text{sc}}(r_{ij}), \tag{S2}$$

where $u_{\text{bond}}(r_{i,i+1})$ and $u_{\text{angle}}(r_{i,i+1}, r_{i+1,i+2})$ are the bonding and angular potential applied for neighboring beads to ensure the connectivity of the chromatin chain.

$$\begin{aligned} u_{\text{bond}}(r_{i,i+1}) &= -\frac{1}{2}KR_0^2 \ln\left[1 - \left(\frac{r_{i,i+1}}{R_0}\right)^2\right], K_b = 30\epsilon, R_0 = 1.5\sigma \\ u_{\text{angle}}(\vec{r}_{i,i+1}, \vec{r}_{i+1,i+2}) &= K_a\left[1 - \cos(\theta - \pi)\right], K_a = 2\epsilon, \cos\theta = \frac{\vec{r}_{i,i+1} \cdot \vec{r}_{i+1,i+2}}{|\vec{r}_{i,i+1}| \cdot |\vec{r}_{i+1,i+2}|} \end{aligned} \tag{S3}$$

$u_c(r_i)$ is a spherical boundary potential applied to each bead to mimic the confinement effect of nuclear envelop.

$$u_c(r_i) = \begin{cases} U_{\text{LJ}}(r_i), r_i \le 2^{1/6}\sigma \\ 0, r_i > 2^{1/6}\sigma \end{cases} \tag{S4}$$

where $U_{\text{LJ}}(r) = 4\epsilon\left[\left(\frac{\sigma}{r}\right)^{12} - \left(\frac{\sigma}{r}\right)^6\right]$ is the Lennard-Jones potential. r_i is the distance between i-th bead and the wall surface. $u_{\text{sc}}(r_{ij})$ is a non-bonded soft-core potential added to each pair formed by beads index i and j to account for the excluded volume effect while allowing finite probability of cross-over of polymer chains.

$$u_{\text{sc}}(r_i) = \begin{cases} 0.5E_{\text{cut}}\left(1 + \tanh\left[\frac{2U_{\text{LJ}}(r_i)}{E_{\text{cut}}} - 1\right]\right), & r_i \le r_{\text{cut}} \\ U_{\text{LJ}}(r_i), & r_{\text{cut}} < r_i \le 2^{1/6}\sigma \\ 0, & r_i > 2^{1/6}\sigma \end{cases} \tag{S5}$$

which corresponds to the Lennard-Jones potential capped off at a finite volume within a replusive core to allow for chain crossing at finite energy cost. $E_{\text{cut}} = 4\epsilon$ and r_{cut} is chosen as the distance at which $U_{\text{LJ}}(r) = 0.5E_{\text{cut}}$.

$U_{\text{intra}}(\mathbf{r})$ is the intra-chromosomal potential applied to genomic loci within the same chromosome, while $U_{\text{inter}}(\mathbf{r})$ is similarly defined but for interactions between loci from different

chromosomes. $U_{\mathrm{Xi}}(\mathbf{r})$ is a weakly attractive potential applied to only one of the two X chromosomes to induce the known X-chromosome inactivation. These terms adopt the following form:

$$U_{\mathrm{intra}}(\mathbf{r}) = \sum_{I} \sum_{i,j \in I} \left[\alpha_{\mathrm{ideal}}(|i-j|) + \alpha_{\mathrm{intra}}(T_i^I, T_j^I)\right] f(r_{ij}) \tag{S6}$$

where I indexes over each chromosome and i and j index over pair of beads on that chromosome. $\alpha_{\mathrm{ideal}}(|i-j|)$ is a function depends only on the sequence separation between two beads i and j. $\alpha_{\mathrm{intra}}(T_i^I, T_j^I)$ depends specifically on the compartment types T_i^I and T_j^I, which can be A, B or C. $f(r_{ij})$ measures the probability of contact formation for two loci separated by a distance of r_{ij}, and its ensemble average corresponds to the contact probability measured in Hi-C experiments. $f(r_{ij})$ adopts the form:

$$f(r_{ij}) = \begin{cases} \frac{1}{2}\left[1 + \tanh\left[\eta(r_c - r_{ij})\right]\right], & r_{ij} \le r_c \\ \frac{1}{2}\left(r_c/r\right)^4, & r_{ij} > r_c \end{cases} \tag{S7}$$

where $r_c = 1.5$ and $\eta = 2.5$. Similarly, we have

$$U_{\mathrm{inter}}(\mathbf{r}) = \sum_{I,J} \sum_{i \in I, j \in J} \alpha_{\mathrm{inter}}(T_i^I, T_j^J) f(r_{ij}), \tag{S8}$$

and

$$U_{\mathrm{Xi}}(\mathbf{r}) = \sum_{i,j \in X\mathrm{i}} w(r_{ij}) = \sum_{i,j \in X\mathrm{i}} \alpha_{X\mathrm{i}}(|i-j|) f(r_{ij}) \tag{S9}$$

The last term in the energy function, $U_{\mathrm{inter}}^{\mathrm{specific}}(\mathbf{r})$, captures specific inter-chromosome interactions beyond the generic compartment-based potential, and adopts the form

$$U_{\mathrm{inter}}^{\mathrm{specific}}(\mathbf{r}) = \sum_{I,J} \sum_{i \in I, j \in J} \alpha_{\mathrm{inter}}^{IJ}(T_i^I, T_j^J) f(r_{ij}) \tag{S10}$$

Mathematical expressions for the various energy terms in $U_{\mathrm{Genome}}(\mathbf{r})$ were designed such that their ensemble averages can be mapped onto combinations of contact frequencies measured in Hi-C. The correspondence between the energy functions and Hi-C measurements allows model parameterization with an efficient maximum entropy optimization algorithm. Specifically, $\alpha_{\mathrm{ideal}}(|i-j|)$, $\alpha_{\mathrm{intra}}(T_i^I, T_j^I)$, $\alpha_{\mathrm{inter}}(T_i^I, T_j^I)$, $\alpha_{X\mathrm{i}}(|i-j|)$, and $\alpha_{\mathrm{inter}}^{IJ}(T_i^I, T_j^J)$ were

tuned to satisfy the following constraints:

$$
\begin{aligned}
\left\langle \sum_{I \neq X} \sum_{i,j \in I} f(r_{ij}) \delta_{|i-j|,s} \right\rangle_{U_{\text{Genome}}(\mathbf{r})} &= \sum_{I \neq X} \sum_{i,j \in I} f_{ij}^{\text{exp}} \delta_{|i-j|,s}, \quad \text{for } s = 1, \cdots, n-1 \\
\left\langle \sum_{I} \sum_{i,j \in I} f(r_{ij}) \delta_{T_i^I,T_1} \delta_{T_j^I,T_2} \right\rangle_{U_{\text{Genome}}(\mathbf{r})} &= \sum_{I} \sum_{i,j \in I} f_{ij}^{\text{exp}} \delta_{T_i^I,T_1} \delta_{T_j^I,T_2}, \quad \text{for } T_1, T_2 \in \{A, B, C\} \\
\left\langle \sum_{I,J} \sum_{i \in I, j \in J} f(r_{ij}) \delta_{T_i^I,T_1} \delta_{T_j^J,T_2} \right\rangle_{U_{\text{Genome}}(\mathbf{r})} &= \sum_{I,J} \sum_{i \in I, j \in J} f_{ij}^{\text{exp}} \delta_{T_i^I,T_1} \delta_{T_j^J,T_2}, \quad \text{for } T_1, T_2 \in \{A, B, C\} \\
\left\langle \sum_{i,j \in X} f(r_{ij}) \delta_{|i-j|,s} \right\rangle_{U_{\text{Genome}}(\mathbf{r})} &= \sum_{i,j \in X} f_{ij}^{\text{exp}} \delta_{|i-j|,s}, \quad \text{for } s = 1, \cdots, n_X - 1 \\
\left\langle \sum_{i \in I, j \in J} f(r_{ij}) \delta_{T_i^I,T_1} \delta_{T_j^J,T_2} \right\rangle_{U_{\text{Genome}}(\mathbf{r})} &= \sum_{i \in I, j \in J} f_{ij}^{\text{exp}} \delta_{T_i^I,T_1} \delta_{T_j^J,T_2}. \quad \text{for } T_1, T_2 \in \{A, B\}
\end{aligned}
\tag{S11}
$$

where $\delta_{T_i^I,T_1}$ is the Kronecker delta function with the following definition:

$$
\delta_{T_i^I,T_1} = \begin{cases} 1, \text{if } T_i^I = T_1 \\ 0, \text{otherwise} \end{cases} \tag{S12}
$$

The angular bracket represents the ensemble average over the Boltzmann distribution $e^{-\beta U_{\text{Genome}}(\mathbf{r})}$ and f_{ij}^{exp} is the corresponding experimental contact frequency.

We applied an iterative algorithm to derive the values for $\alpha_{\text{ideal}}(|i-j|)$, $\alpha_{\text{intra}}(T_i^I, T_j^I)$, $\alpha_{\text{inter}}(T_i^I, T_j^I)$, $\alpha_{X\text{i}}(|i-j|)$, and $\alpha_{\text{inter}}^{IJ}(T_i^I, T_j^J)$ that enforce the constraints defined in Eq. S11. As shown in Ref. 1, while the model was parameterized only with population Hi-C data, it succeeded in reproducing a variety of observations from imaging studies. For example, A/B compartments were shown to occupy distinct nuclear regions, with B compartments preferentially at the periphery. The model further captures the formation of chromosome territories, the clustering of centromeric regions, and the radial position of individual chromosomes.

Estimating the Surface Tension of Simulated Droplets

The surface tension (γ) of simulated droplets were estimated using the expression from equipartition theorem as $\gamma = k_{\text{B}}T/\langle u^2 \rangle$. $\langle u^2 \rangle$ measures the fluctuation of droplet size and

was computed as follows. For every simulated configuration, we identified the droplets and calculated the size of individual droplets as $R_g^i = \sqrt{\frac{1}{N}\sum_{n=1}^{N} |\mathbf{r}_n - \mathbf{r}_{\text{com}}|^2}$, where N is the number of nucleolar particles in the droplet. $\mathbf{r}_n$ is the Cartesian coordinate of nucleolar particle n and $\mathbf{r}_{\text{com}}$ is the center of mass of the droplet. We then estimated the fluctuation as

$$\langle u^2 \rangle = \frac{\sum_{i=1}^{M}(R_g^i - \bar{R}_g)^2}{M}, \tag{S13}$$

where $\bar{R}_g$ is the mean value and M is the number of simulated configurations.

The fluctuation $\langle u^2 \rangle$ estimated using all the droplets found in the 12 trajectories was $0.035\ \sigma^2$. Using $\sigma = 0.25\ \mu$m, we have $\gamma = 1.9 \times 10^{-6} N \cdot m^{-1}$ which agrees well with the experimental value [2]. An example trajectory of the R_g with respect to the simulation time is shown in Fig. S8B.

Details for Simulations with a Dissolved Chromatin Network

To directly probe the impact of the chromatin network on phase separation, we performed additional simulations with a dissolved chromatin network. Specifically, we dissolved the chromatin network by removing polymeric connectivity and the bonding potential between neighboring chromatin beads. Non-bonded interactions between chromatin beads were also removed. All other setups, including protein-protein and protein-chromatin interactions as well as the boundary potential mimicking nuclear confinement [1], were kept the same. We carried out a total of twelve 20-million-step independent simulations starting from an equilibrated genome structure without bead-bead connections and randomly distributed nucleolar particles for studying phase separation dynamics (Fig. S3). In the free energy calculations, we used the same setup for umbrella sampling and temperature replica exchange as the original system. The simulations were initialized from a two-droplet configuration and lasted for twelve million steps for each umbrella window.

Identifying the Neck Region between Two Merging Droplets

To quantify the coalescence dynamics between droplets, we measured the radius of their neck region as follows. For each simulated configuration, we first determined the principal

axes as eigenvectors of the covariance matrix

$$\mathbf{C} = \begin{bmatrix} \mathbf{x}\mathbf{x}^{\mathbf{T}} & \mathbf{x}\mathbf{y}^{\mathbf{T}} & \mathbf{x}\mathbf{z}^{\mathbf{T}} \\ \mathbf{y}\mathbf{x}^{\mathbf{T}} & \mathbf{y}\mathbf{y}^{\mathbf{T}} & \mathbf{y}\mathbf{z}^{\mathbf{T}} \\ \mathbf{z}\mathbf{x}^{\mathbf{T}} & \mathbf{z}\mathbf{y}^{\mathbf{T}} & \mathbf{z}\mathbf{z}^{\mathbf{T}} \end{bmatrix} \tag{S14}$$

Here, $\mathbf{x}$ is the row vector of the shifted Cartesian coordinates along the $x-$axis for individual particles $\mathbf{x} = (x_1 - \bar{x}, x_2 - \bar{x}, \cdots, x_N - \bar{x})$, where $\bar{x} = \frac{1}{N}\sum_i^N x_i$. $\mathbf{y}$ and $\mathbf{z}$ are similarly defined along the $y-$ and $z-$axis. All particles from both droplets were used in computing the covariance matrix. The principal axis with largest (smallest) eigenvalue corresponds to the direction with most (least) significant particle fluctuation and is noted as $\vec{v_l}$ ($\vec{v_s}$).

We projected all nucleolar particles onto the principal axes, and computed the width of the dumbbell as the difference between the maximum and minimum value along the $\vec{v_s}$ axis. As shown in Fig. S13, the resulting width profile along $\vec{v_l}$ exhibits double peaks corresponding to the central position of each droplet. The neck region lies at the minimum of the profile and the corresponding value provides a measure of the neck radius.

Identifying Clusters along Phase Separation with the DBSCAN Algorithm

We applied the DBSCAN (Density-Based Spatial Clustering of Applications with Noise) algorithm [3] to identify spatial clusters formed by nucleolar particles. DBSCAN is a density-based clustering algorithm, which finds "hot regions" or clusters of high spatial density followed by their gradual expansion with neighboring samples. There are two key parameters in this algorithm, which correspond to the maximum distance between two samples (r_{max}) for them to be considered as neighbors and the minimum number of samples (N_{min}) required for cluster identification. The values for these parameters are system dependent and we used $r_{\text{max}} = \sigma$ and $N_{\text{min}} = 5$. Varying the values of the two parameters within reasonable ranges has minimal effect on the results present in the manuscript.

Mapping the Reduced Time Unit to Real Time

We mapped the reduced time unit used in simulations onto the physical unit by matching the diffusion coefficient from simulations with that in the nucleus. Specifically, from the Stokes-Einstein (SE) equation, the experimental diffusion coefficient can be calculated as

$D = \frac{k_{\mathrm{B}}T}{6\pi\eta r}$, where η is the viscosity and r is the radius of spherical beads. The exact value of nucleoplasmic viscosity is controversial and has been estimated to values that differ by orders of magnitude. For example, fluorescence recovery after photobleaching (FRAP) [4] and fluorescence correlation spectroscopy [5] experiments that track protein diffusion have provided estimations for the nucleoplasmic viscosity on the order of 10^{-3} Pa·s to 10^{-2} Pa·s. On the other hand, studies that tracked the diffusion of Cajal bodies in the intranuclear region of Hela cells [6] or the diffusion of microinjected nanoparticles [7] has estimated the nucleoplasmic viscosity to be 10^{-1} Pa·s to 10^{2} Pa·s. The wide variation of the experimental measurements might be due to the heterogeneity of the cellular environment [2]. In this study, we used an intermediate estimation of the nucleoplasmic viscosity as 10^{-2} Pa·s. At this value, our simulated timescale for protein exchange (Fig. S8) agrees well with measurements from FRAP experiments [8].

The diffusion coefficient in our simulations can be estimated from the fluctuation-dissipation theorem [9] as $D = \frac{k_{\mathrm{B}}T}{\zeta}$, where the friction coefficient $\zeta = \frac{m}{\gamma}$. Since the the damping coefficient γ was set as $10\tau_B$ in the Langevin dynamics, we have $D = \frac{k_{\mathrm{B}}T}{m} \cdot \gamma = \frac{10k_{\mathrm{B}}T}{m} \cdot \tau_B = 10 \cdot \frac{\sigma^2}{\tau_B}$. τ_B is the reduced time unit, and σ is the diameter of the chromatin bead. Assuming a nucleus of 5 μm in radius, σ can be estimated to be $\sim 0.25\mu m$ given the size of the spherical confinement ($R_{\mathrm{N}} = 19.7\sigma$) used in simulations. Combining the two expressions for the diffusion coefficient, we arrive at the expression $\tau_B = \frac{10\sigma^2 \cdot 6\pi\eta r}{k_{\mathrm{B}}T} = \frac{30\pi\eta\sigma^3}{k_{\mathrm{B}}T} \approx 3.6s$ and the simulation timestep $dt = 0.008\tau_B = 0.028s$.

Estimating Radial Distribution of Chromosomes

The radial probability density distribution $\rho(r)$ presented in Fig. 2D of the main text was calculated based on the following expression

$$\rho_i(r) = \frac{\langle N_i(r) \rangle_{U_{\mathrm{Model}}}(\mathbf{r})}{4\pi r^2 \Delta r \cdot N_i} \tag{S15}$$

where $N_i(r)$ is the number of beads of type i located in the spherical shell from radial distance r to $r + \Delta r$. The angular bracket $\langle \cdot \rangle$ stands for the ensemble average over all simulated configurations. N_i is the total number of beads of type i.

Simulating Phase Separation with a Tumor Genome Model

To validate the generality of our results and mechanisms with respect to cell types, we parameterized an independent diploid genome model to carry out additional simulations of phase separation. Hi-C data generated from clinical tissue samples with colorectal cancer were used for model parameterization [10]. As shown in Fig. S17A and B, the average contact frequencies at the compartmental level calculated using tumor Hi-C data differ significantly from those determined using the GM12878 data, i.e., the one used in the main text. We applied the same algorithm introduced in Ref. 1, which was used to derive the genome model presented in the main text, to optimize the tumor genome model.

The 3D organization of the tumor genome differs significantly from that of GM12878 cells. While chromosomal territories are still clearly present (Fig. S17C), the preferential localization of B compartments at the nuclear periphery is lost (Fig. S17E). Furthermore, the radial positions of tumor chromosomes correlate poorly with that of GM12878 cells (Fig. S17D). The dramatic differences in the genome organization of the two cell types render the tumor genome model a good test system for validating the robustness of the mechanism of nucleoli formation.

With the newly optimized interactions for the tumor genome, we introduced nucleolar particles to the system. The number of nucleolar particles and the strength of nucleolar particle-nucleolar particle and nucleolar particle-chromatin interactions were kept the same as those used in the main text. Following the same protocol as in the main text, we performed 12 independent 20-million-step-long simulations to probe phase separation. The distribution for the number of droplets recorded at the end of these simulations is shown in Fig. S17F. The multi-droplet state is again favored. Notably, the average number of droplets observed in tumor simulations is higher than that for GM12878 cells. Cancer nuclei are indeed known to have higher nucleoli numbers [11–13]. A possible reason for this increase could be due to the reduced inter-chromosome interactions in tumor cells (Fig. S17B). Chromosomes are less constrained with weaker interactions, and the entropic penalty for bringing them into close contact upon droplet coalescence is conceivably higher.

Therefore, the chromatin network for the tumor genome, while differing significantly from that for GM12878 cells, stabilizes the formation of multiple droplets. Furthermore, our model succeeds in capturing the qualitative change in nucleoli number upon tumorigenesis.

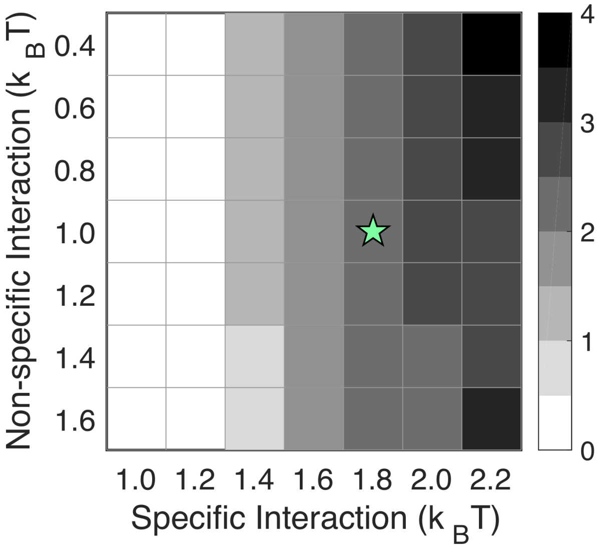

FIG. S1: **Average number of droplets at various combinations of specific and non-specific interactions between nucleolar particles and chromatin.** For each parameter set, we followed the same protocol as in the main text to simulate phase separation. For example, twelve independent 20-million-step-long trajectories were performed, and the droplet number at the end of each simulation trajectory was recorded to compute the averages. The star indicates the value used in simulations presented in the main text. From the above figure, it is evident that as soon as the strength of specific interactions becomes strong enough to promote phase separation, the multi-droplet state emerges. At the strength of 1.4 k_BT, though droplets begin to emerge in some simulations, there are still significant simulations without phase separation. The average number of droplets is, therefore, less than one. Droplets appear in all simulations at 1.6 k_BT, and many trajectories produce multiple droplets. We decided to use 1.8 k_BT, which leads to multiple droplets in most simulations. Furthermore, the surface tension of the resulting droplets is comparable to the experimental value as well. The non-specific interaction was chosen as 1.0 kT, but as the phase diagram shows, it has little impact on the results.

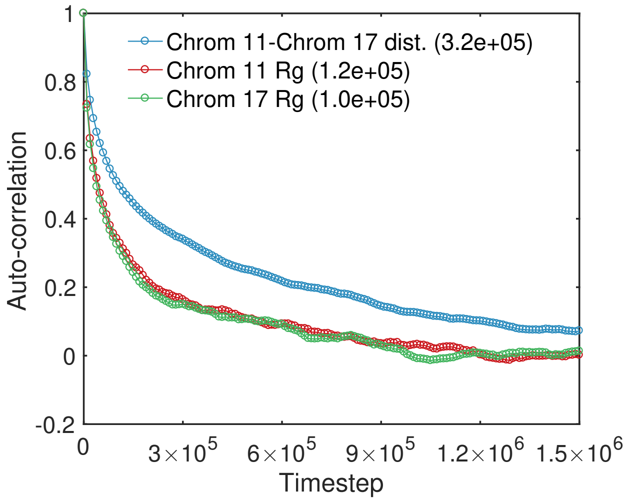

FIG. S2: **The correlation time of representative collective variables is much shorter than the length of simulated trajectories, supporting their equilibration.** Autocorrelation function of the center of mass distance between chromosome 11 and 17 (blue), the radius of gyration of chromosome 11 (red) and chromosome 17 (green) as a function of time. Numbers in the parentheses are the value of the characteristic decay timescale (τ) fitted using the function $\exp(-t/\tau)$.

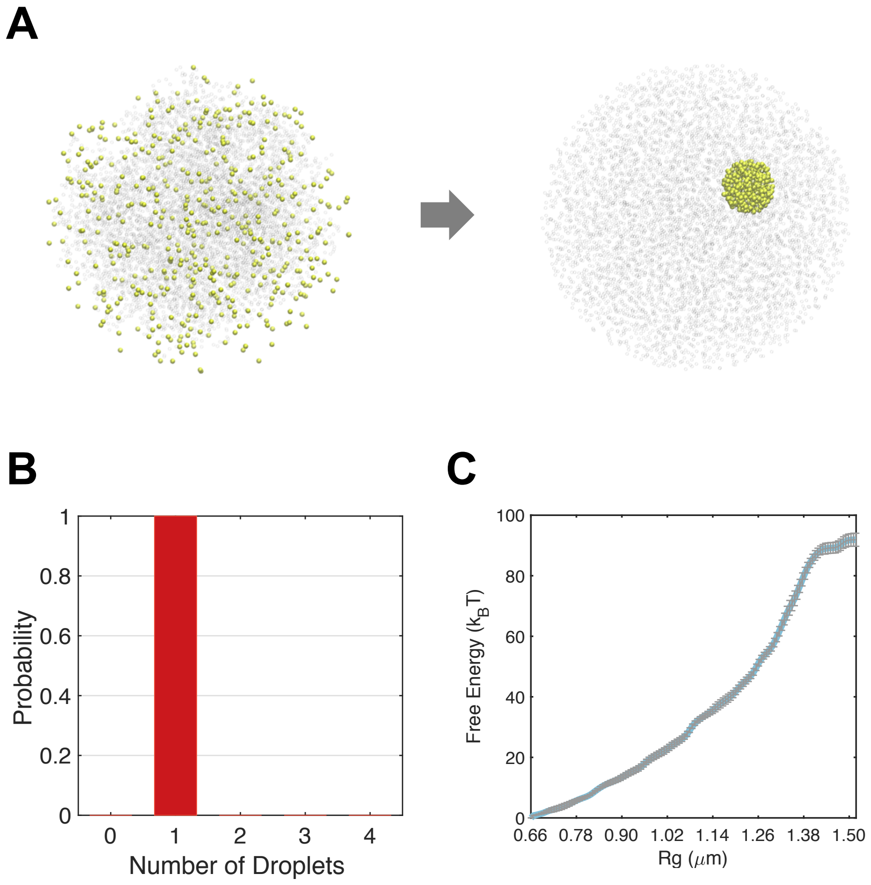

FIG. S3: **Dissolving the chromatin network leads to single droplet formation.** (A) Representative initial (left) and final (right) configurations obtained from dynamical simulations of the nucleus model with a dissolved chromatin network, with nucleolar particles in yellow and the rest of the genome in grey. (B) Probability distribution of the number of droplets observed at the end of simulation trajectories. (C) Free energy profile as a function of R_g for simulations performed with a dissolved chromatin network. Error bars were calculated as standard deviation of the mean. See text *Section:Details for Simulations with a Dissolved Chromatin Network* for further discussion.

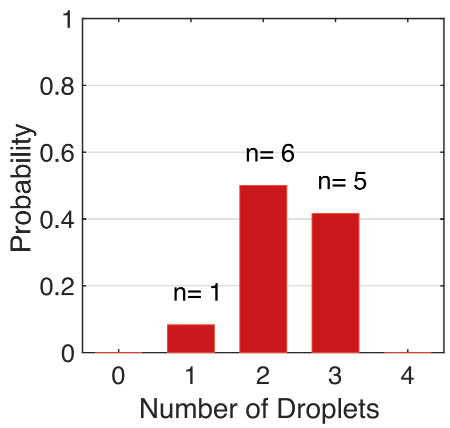

FIG. S4: **Probability distribution of the number of droplets observed at the end of simulation trajectories initialized with different chromosome conformations.** The simulations presented in Fig. 2B of the main text were initialized with the same configuration but different random velocities. We carried out 12 additional 20-million-step-long simulations to further evaluate the robustness of our results with respect to the initial configuration. These new simulations were initialized with different configurations prepared as following. We first collected 12 uncorrelated sets of chromosome conformations from a long simulation trajectory of the genome-only model at equal time intervals. For each set of conformations, we then introduced nucleolar particles and relaxed the resulting structures following the same procedure detailed in the *Methods Section* of the main text. The distribution for the number of droplets recorded at the end of these simulations is quantitatively comparable to that shown in Fig 2B. Therefore, results presented in the main text are not sensitive to the initial conformations of chromosomes.

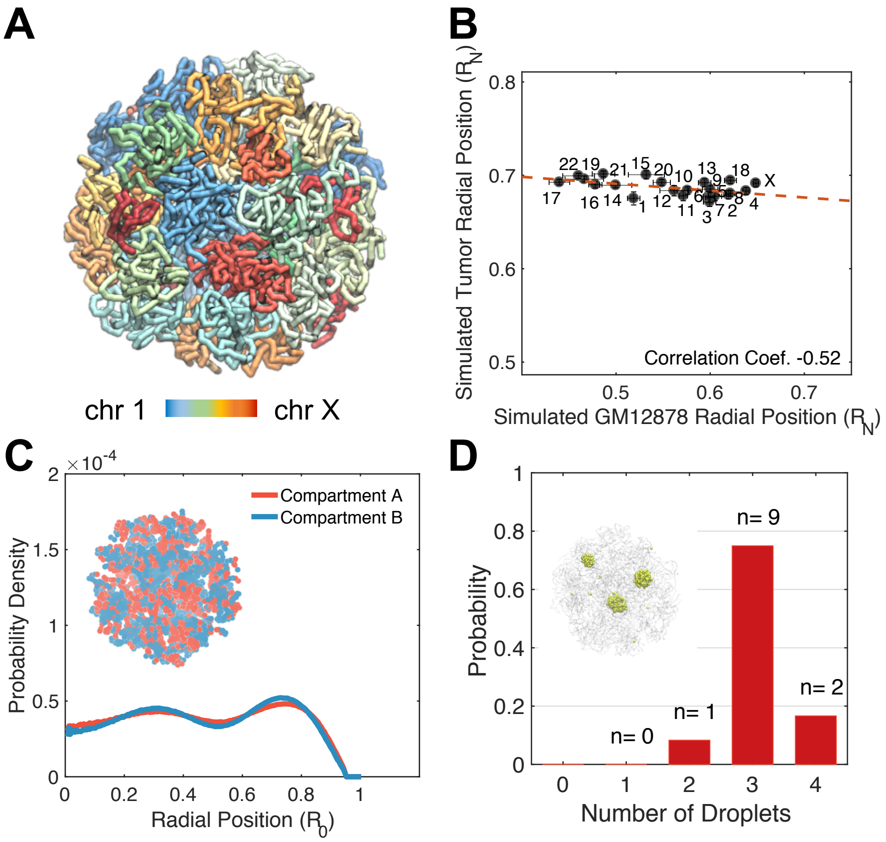

FIG. S5: **The genome organization obtained after removing Hi-C optimized inter-chromosome interactions, while differs significantly from the one presented in the main text, supports the stability of the multi-droplet state.** We followed the same protocols as those used to produce Fig. 2 of the main text to carry out 12 additional independent 20-million-step-long simulations with α_{inter} defined in Eq. S8 and Eq. S10 set to 0. (A) Representative configuration of the genome that illustrates the formation of chromosome territories. (B) Correlation between chromosome radial positions obtained using the perturbed model and the original results presented in the main text. Error bars correspond to the standard deviation of the 12 mean values estimated using individual simulation trajectories. Homologous chromosomes were averaged together. R_{N} is the radius of the nucleus used in polymer simulations. (C) Radial distributions of A/B compartments. An example genome configuration is shown as the inset, with the two compartments colored in red (A) and blue (B) respectively. (D) Probability distribution of the number of droplets observed at the end of simulation trajectories.

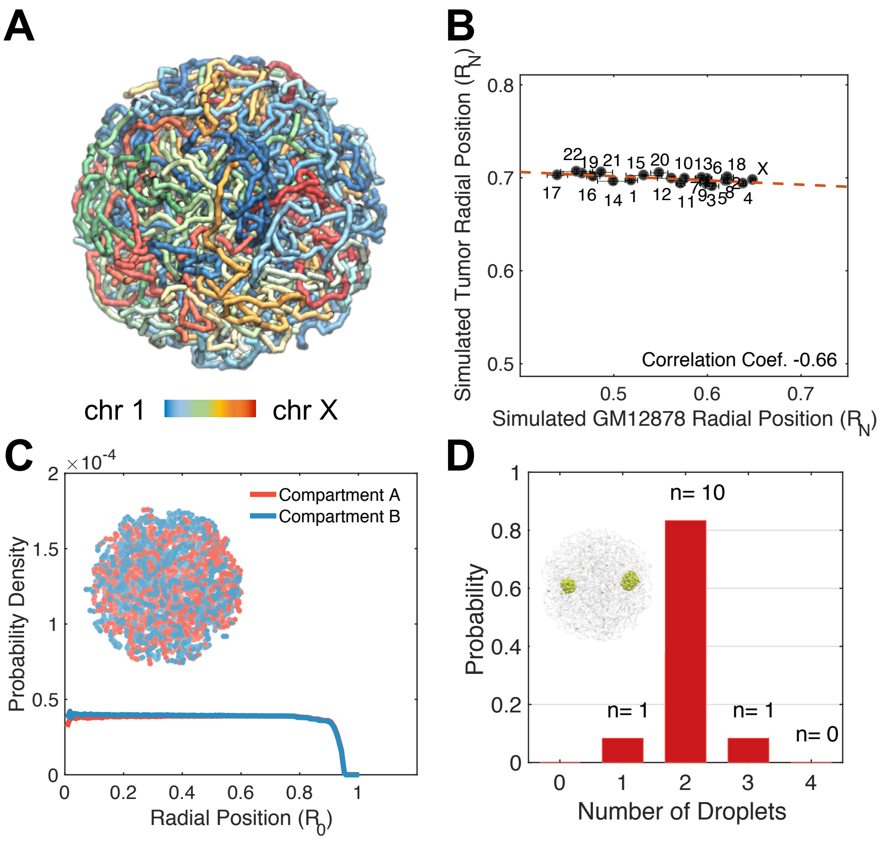

FIG. S6: **The genome organization obtained after removing Hi-C optimized intra- and inter-chromosome interactions, while differs significantly from the one presented in the main text, supports the stability of the multi-droplet state.** We followed the same protocols as those used to produce Fig. 2 of the main text to carry out 12 additional independent 20-million-step-long simulations with α_{inter} defined in Eq. S8 and Eq. S10 and α_{intra} defined in Eq. S6 all set to 0. (A) Representative configuration of the genome that illustrates the formation of chromosome territories. (B) Correlation between chromosome radial positions obtained using the perturbed model and the original results presented in the main text. Error bars correspond to the standard deviation of the 12 mean values estimated using individual simulation trajectories. Homologous chromosomes were averaged together. R_{N} is the radius of the nucleus used in polymer simulations. (C) Radial distributions of A/B compartments. An example genome configuration is shown as the inset, with the two compartments colored in red (A) and blue (B) respectively. (D) Probability distribution of the number of droplets observed at the end of simulation trajectories.

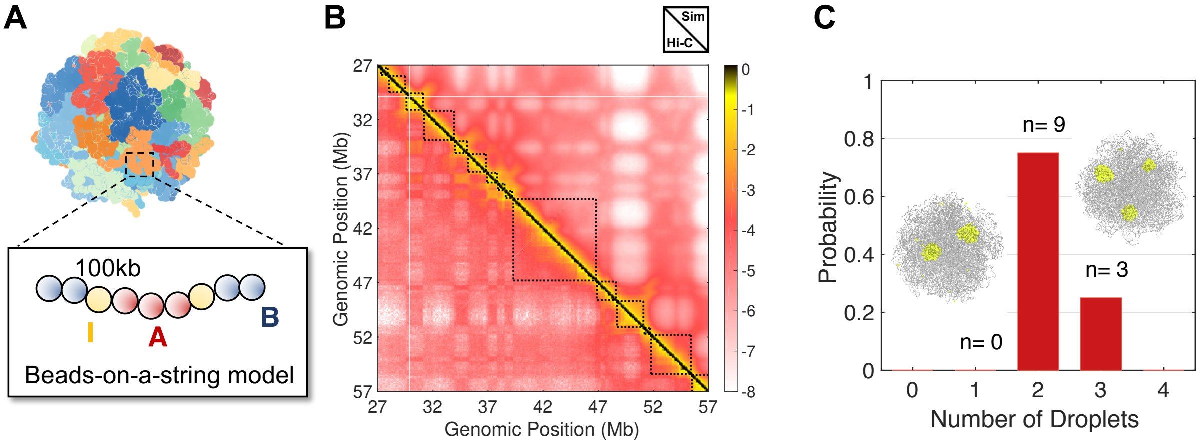
A
100kb
I
A
B
Beads-on-a-string model
B
Sim
Hi-C
Genomic Position (Mb)
Genomic Position (Mb)
27
32
37
42
47
52
57
0
-1
-2
-3
-4
-5
-6
-7
-8
C
Probability
Number of Droplets
n= 9
n= 3
n= 0
0
0.2
0.4
0.6
0.8
1
0
1
2
3
4

FIG. S7: **A 100kb resolution genome model supports the stability of the multi-droplet state.** (A) Overview of the 100kb resolution model. Similar to the model presented in the main text, chromosomes are represented as strings of coarse-grained beads, each one of which represents a genomic region of 100kb in length. Each bead was further labeled as compartment A, B, or I. As detailed in Ref. 10, compartment I differs from both A and B with unique histone modification and DNA methylation patterns. The energy function of this model is similar to $U_{\text{genome}}(\mathbf{r})$ defined in the Supplementary Material. One notable difference is our differentiation of interactions within individual topologically associating domains (TAD) from those across different TADs. Together with the model's higher resolution, this differentiation allows it to recapitulate high-resolution structural motifs of the genome seen in Hi-C data. Due to the computational cost with increased resolution, we only modeled a single copy of each chromosome, i.e., the haploid genome. The exact expression of the energy function can be found in Ref. 10. Parameters in the energy function were again derived using the maximum entropy optimization algorithm based on the Hi-C data for normal human colon tissue samples processed at the 100kb resolution. (B) Comparison between experimental (bottom left) and simulated contact map (top right) for a representative genomic region from chromosome 1 (27Mb to 57Mb). Dotted blocks along the diagonal are TAD boundaries determined using experimental data with the software TADbit [14]. (C) Probability distribution of the number of droplets observed at the end of twelve independent 12-million-step-long simulation trajectories. Insets are representative snapshots for the two-droplet and the three-droplet states. We followed the same simulation protocols as in the main text to simulate phase separation with the 100kb model. We introduced the same number of nucleolar particles into the model as what we have in the 1Mb model, but scaled the size of each protein bead similarly according to the procedure in the *Methods: Estimating the Size and Number of Nucleolar Particles* Section in the main text. All the interaction strengths for protein-protein and protein-chromatin interactions were set to be the same as those in the 1Mb model.

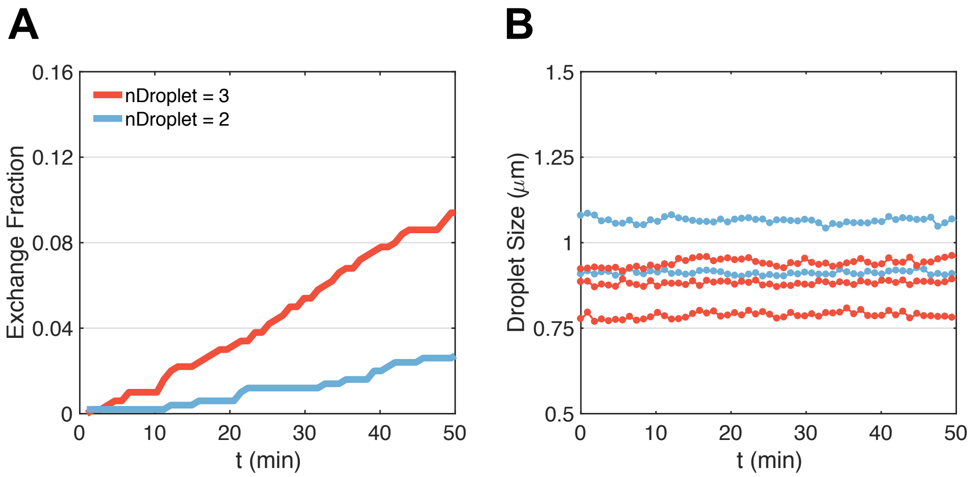

FIG. S8: **Nucleolar particles undergo dynamic exchange with surrounding nucleoplasm while maintaining the droplet size.** (A) Fraction of nucleolar particles that have left the original droplet as a function time for the two representative trajectories with 3 (red) and 2 (blue) droplets. (B) Droplet sizes calculated as the radius of gyration (R_g) as a function of time along the two trajectories shown in (A).

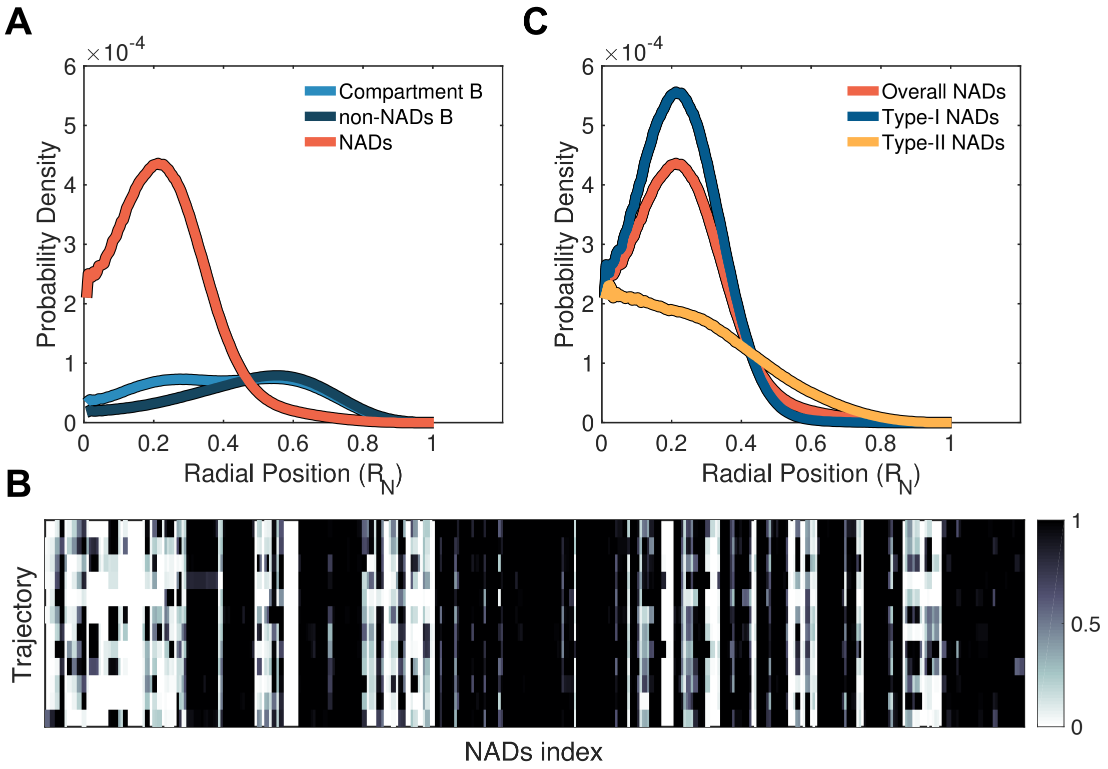

FIG. S9: **NADs are in general closer to nuclear interior than other heterochromatin but a significant fraction of them localizes towards the nuclear envelope.** (A) Radial distribution profile of compartment B loci, compartment B loci that are not identified as NADs, and NADs. (B) Probability of each NAD (x-axis) to be in close contact with simulated droplets (nucleoli) in different trajectories (y-axis). Two types of NADs are evident. We identified type I NADs as those that show high probability of nucleoli association in all trajectories with an average probability larger than 0.5. The rest is classified as type II NADs. (C) Radial distribution profiles of different NADs support their differential nuclear localization. R_N is the radius of the nucleus used in polymer simulations.

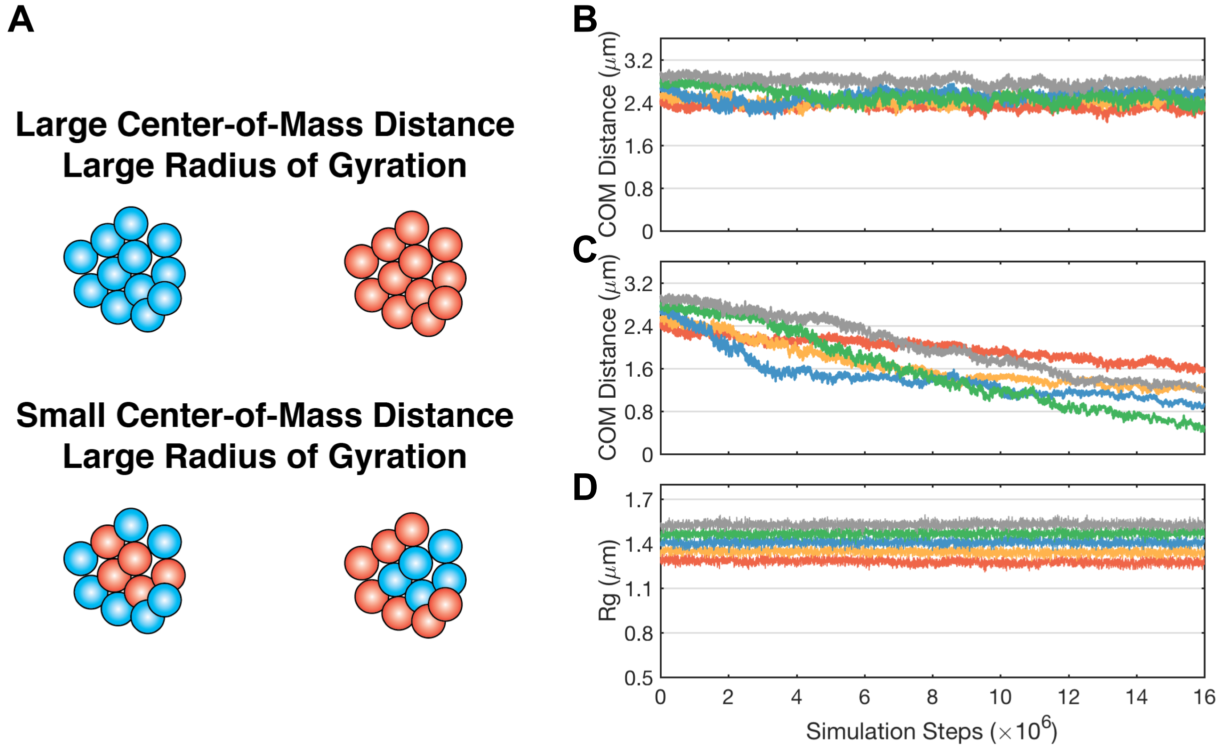

FIG. S10: **The radius of gyration (R_g) succeeds at driving the separation of droplets.** (A) Illustration of the problem of using a fixed set of nucleolar particles to compute the center of mass (COM) distance between two droplets. As the particles from two droplets exchange as shown in the bottom panel, the COM distance between red and blue particles will decrease even when the two droplets are far apart from each other. (B,C,D) The COM distance obtained from post-analysis of simulation trajectories with the nucleolar particles in each droplet identified on the fly (B), the COM distance using a fixed set of nucleolar particles in each droplet identified at the beginning of simulations, and R_g as a function of simulation time. Different colors represent different trajectories.

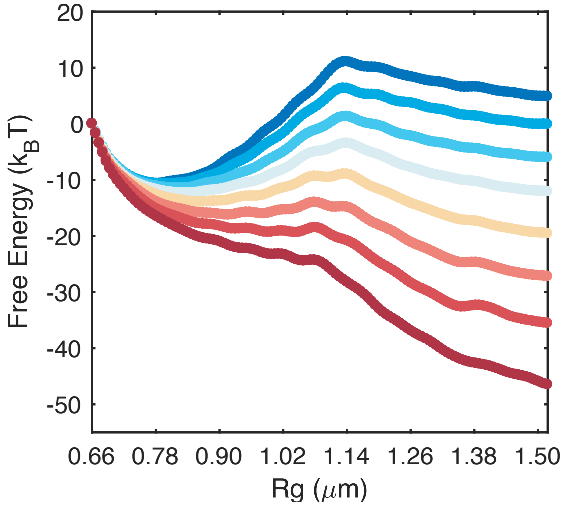

FIG. S11: Free energy profiles as a function of R_g calculated at temperatures from 1.00 to 1.14 with an increment of 0.02. The temperature increases as the color varies from blue to red.

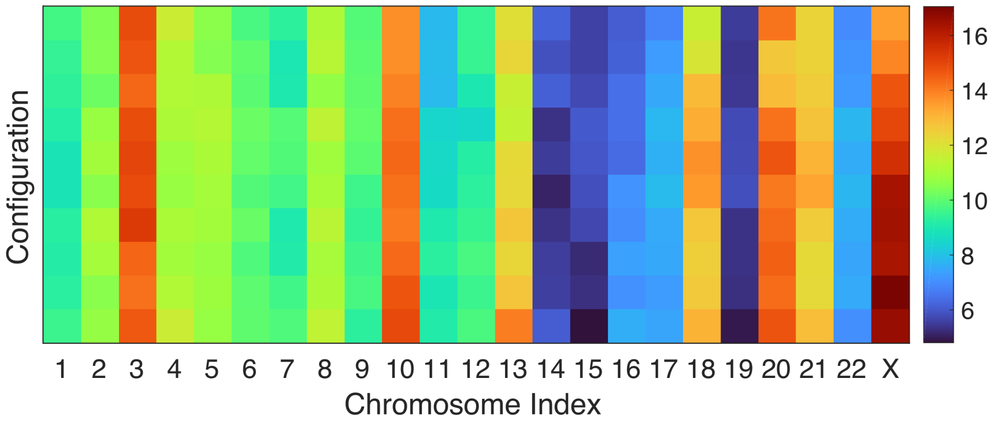

FIG. S12: Variation of the center of mass distance between chromosomes (x-axis) and one of the simulated droplets (nucleoli) in different configurations (y-axis) used to initialize the dynamical simulations in Fig. 4A of the main text. The unit of distance is σ. The noticeable differences among configurations support significant rearrangement of chromosome positions.

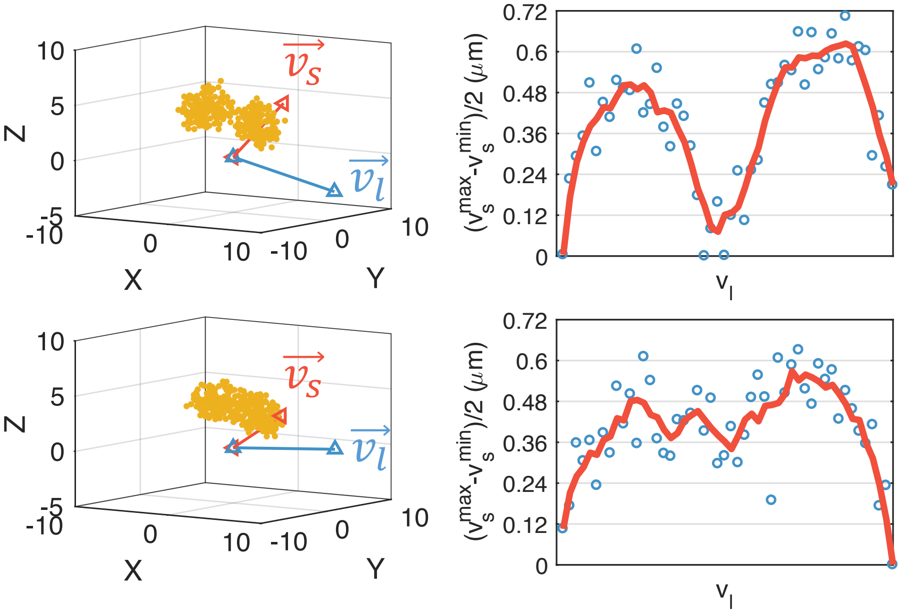

FIG. S13: **Identifying the neck region between two merging droplets from principal axes.** Left Panel: representative configurations at the initial (top) and final (bottom) stage of droplet coalescence shown in the original Cartesian space. $\vec{v_l}$ ($\vec{v_s}$) is the principal axis with largest (smallest) eigenvalue and corresponds to the direction with most (least) significant particle fluctuation. Right Panel: The width profiles obtained after projecting nucleolar particles onto the principal axes. See text *Section:Identifying the Neck Region between Two Merging Droplets* for further discussions.

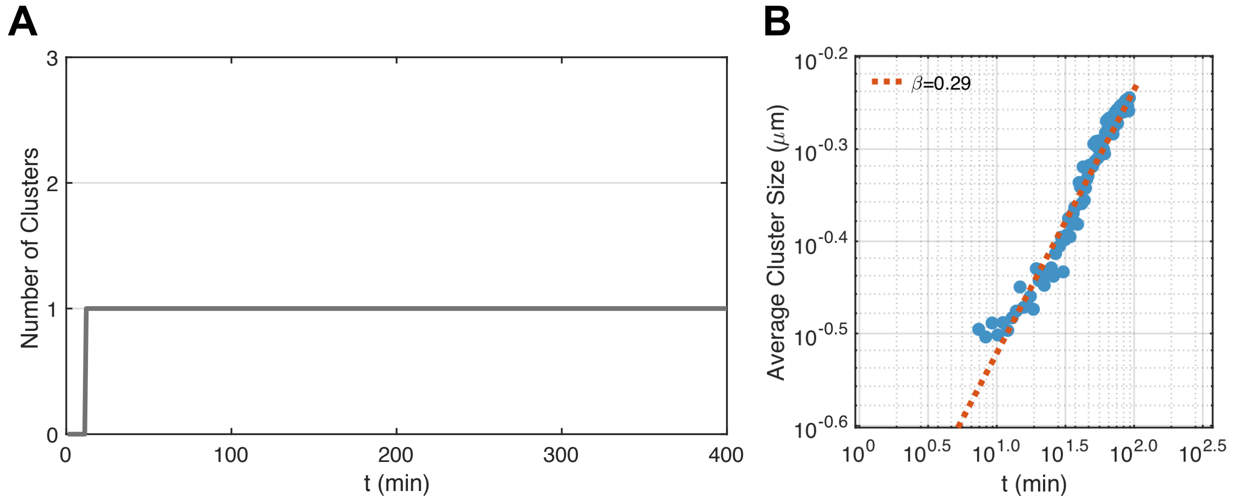

FIG. S14: **Coarsening dynamics for phase separation with a dissolved chromatin network.** (A) Time evolution of the number of clusters observed along a simulated trajectory. (B) Power-law scaling of the average cluster size as a function of time. See text *Section:Details for Simulations with a Dissolved Chromatin Network* for further discussion.

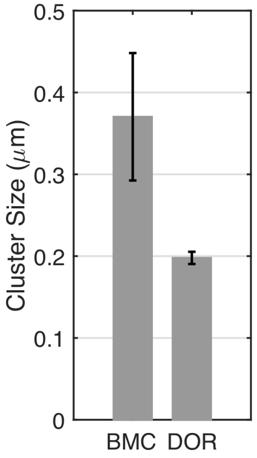

FIG. S15: Bar plot for the average size of the clusters that undergo Brownian motion-induced coalescence (BMC) and diffusion-limited Ostwald ripening (DOR) pathways. The statistics were done on 92 BMC events and 13 DOR events. Error bars correspond to standard deviations of all clusters that undergo each pathway.

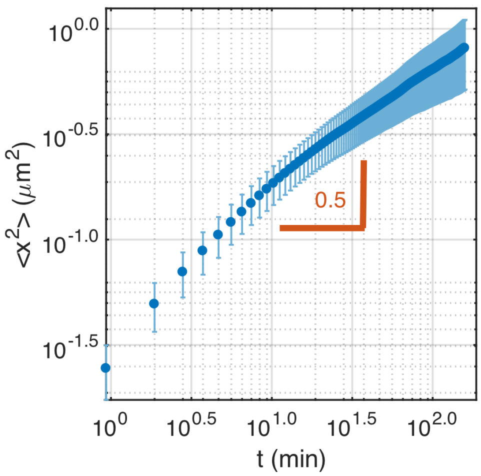

FIG. S16: **Clusters exhibit sub-diffusive motion.** Mean-square displacement (MSD) of the center-of-mass of clusters as a function of time. Error bars correspond to standard deviations of the MSD for all clusters across all 12 independent simulated trajectories.

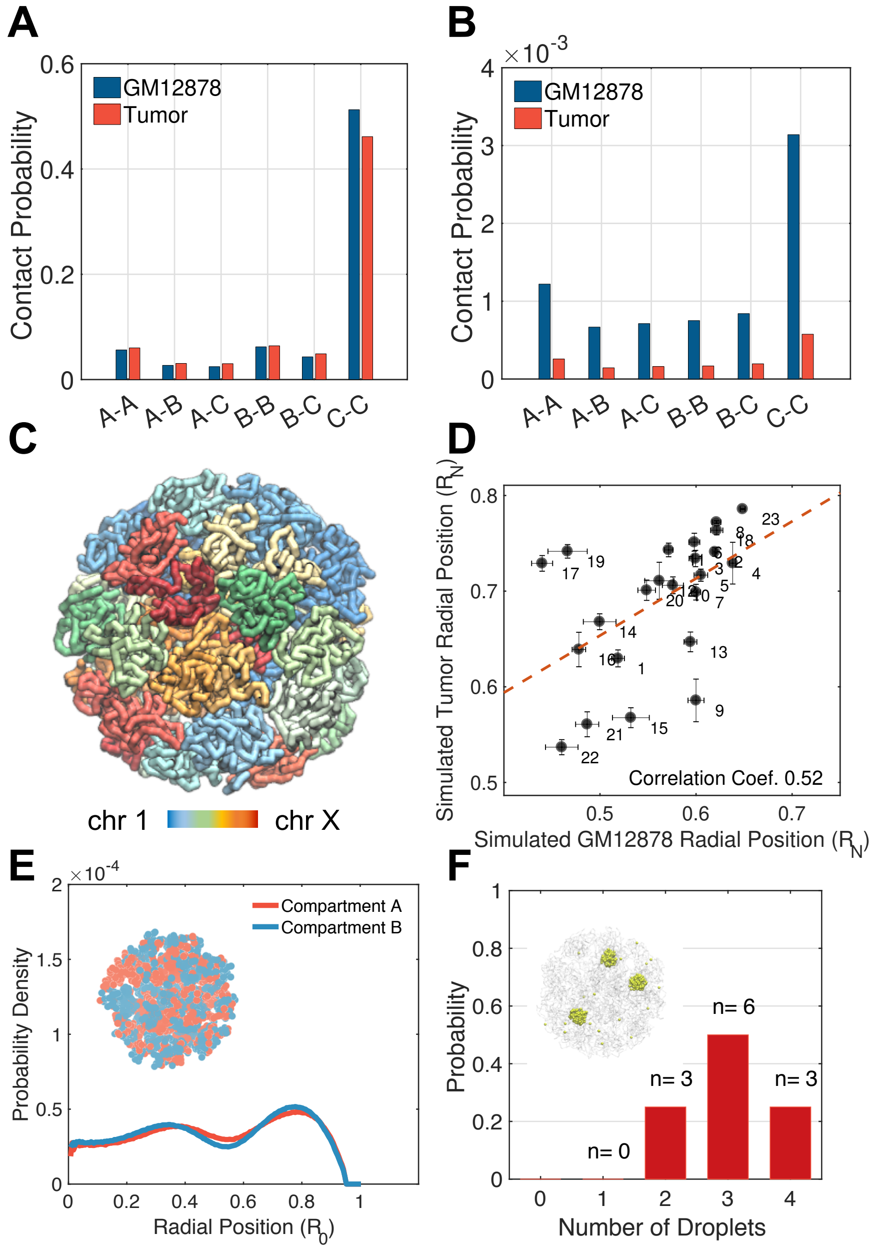
A
Contact Probability
GM12878
Tumor
A-A
A-B
A-C
B-B
B-C
C-C
B
×10-3
C
chr 1
chr X
D
Simulated Tumor Radial Position (RN)
Simulated GM12878 Radial Position (RN)
Correlation Coef. 0.52
E
×10-4
Compartment A
Compartment B
Probability Density
Radial Position (R0)
F
Probability
Number of Droplets
n= 0
n= 3
n= 6
n= 3

FIG. S17: **Tumor genome organization, while differs significantly from the one presented in the main text, supports the stability of the multi-droplet state.** See text *Simulating Phase Separation with a Tumor Genome Model* for details. (A, B) Average intra (A) and inter (B) chromosome contact probabilities between various compartments estimated using Hi-C data for GM12878 (blue) and tumor (red) cells. (C) Representative configuration of the genome that illustrates the formation of chromosome territories. (D) Correlation between chromosome radial positions obtained using simulations of the GM12878 and tumor genome model. Error bars correspond to the standard deviation of the mean values estimated using individual simulation trajectories. Homologous chromosomes were averaged together. R_{N} is the radius of the nucleus used in polymer simulations. (E) Radial distributions of A/B compartments. An example genome configuration is shown as the inset, with the two compartments colored in red (A) and blue (B) respectively. (F) Probability distribution of the number of droplets observed at the end of simulation trajectories.

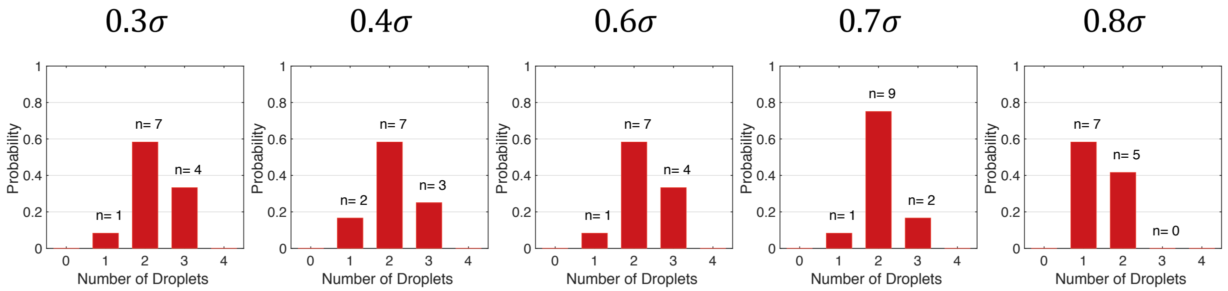

FIG. S18: **The stability of the multi-droplet state is robust with respect to nucleolar particle size.** To examine the dependence of simulation results on the size of nucleolar particles, we altered its value ($\sigma_{\rm P}$) from 0.3σ to $0.3\sigma, 0.4\sigma, 0.6\sigma, 0.7\sigma, 0.8\sigma$. All other parameters in the model were kept the same. For each value of $\sigma_{\rm P}$, we followed the same protocols as those used to produce Fig. 2 of the main text to carry out 12 independent 20-million-step-long simulations. The number of droplets formed at the end of each simulation was then recorded to compute the corresponding probability distributions shown above. As can be seen in this figure, the multi-droplet state appears for all parameter values. It is also more populated than the mono-droplet state except for $\sigma_{\rm P} = 0.8\sigma$. However, at $\sigma_{\rm P} = 0.8\sigma$, the estimated nucleoli size would be out of the reasonable range estimated from microscopic images.

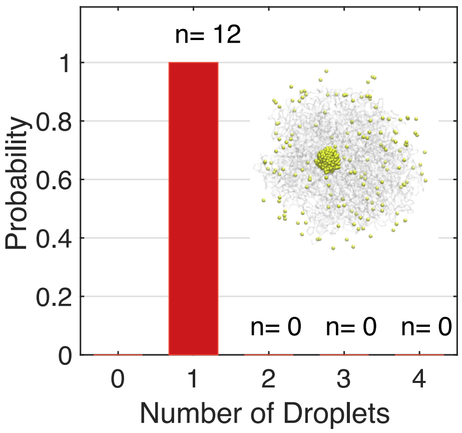

FIG. S19: **Removing nucleolar particle-chromatin interactions destabilizes the multi-droplet state.** To examine the dependence of simulation results on the interaction between nucleolar particles and chromatin, we set the specific interaction strength, i.e., ϵ defined in Eq. 1 of the main text, to 1.0 $k_B T$. The nucleolar particle-nucleolar particle interaction and other parameters for interactions among chromosomes were kept the same. We followed the same protocols as those used to produce Fig. 2 of the main text to carry out 12 independent 20-million-step-long simulations. The number of droplets formed at the end of each simulation was then recorded to compute the corresponding probability distributions shown above.

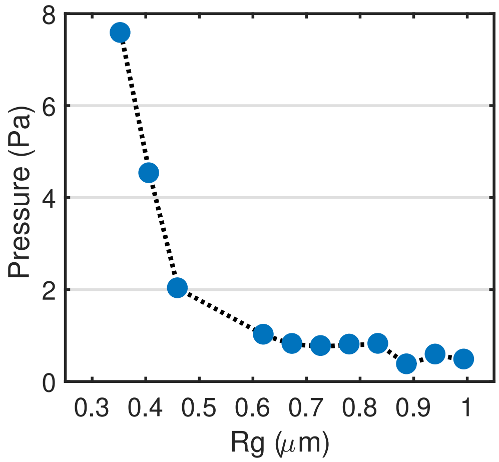

FIG. S20: **Pressure as a function of the droplet size.** We computed the pressure for all the droplets formed in phase separation simulations using the expression introduced by Lion and Allen [15]: $P(\vec{r}) = \frac{1}{3\Omega}\left\langle \sum_{i=1}^{N} \frac{|\vec{p_i}|^2}{m_i}\Lambda_i + \sum_{i=1}^{N-1}\sum_{j>i}(\vec{f_{ij}}\cdot\vec{r_{ij}})l_{ij}\right\rangle$. Here, Ω is the volume of the region of interest, centred on $\vec{r}$, Λ_i is the unity if particle i lies within the volume Ω, and zero otherwise. l_{ij} is the fraction $(0 \leq l_{ij} \leq 1)$ of the line joining particles i and j that lies within Ω. $\vec{r_i}$ and $\vec{r_j}$ are the positions of particles i and j, $\vec{r_{ij}} = \vec{r_i} - \vec{r_j}$, and $\vec{f_{ij}}$ denotes the force exerted on particle i by particle j. $\vec{p_i}$ and m_i are the momentum and mass of particle i. The bracket $\langle\cdot\rangle$ represents the ensemble average over independent configurations with droplet size that are within $\pm 0.0534\mu m$ of the presented values. For simplicity, we approximated the first term as $N_d k_B T/\Omega = \rho_d k_B T$ using the equal partition theorem $\frac{|\vec{p_i}|^2}{m_i} = m_i|\vec{v_i}|^2 = 3k_BT$, where N_d and ρ_d are the number of particles and density of droplets. The dependence of pressure on droplet size differs from the result obtained by Zhang et al. [16], where the pressure was found to increase for larger droplets. There, the authors used a cross-linked network and repulsive interactions between phase-separating agents and polymers.

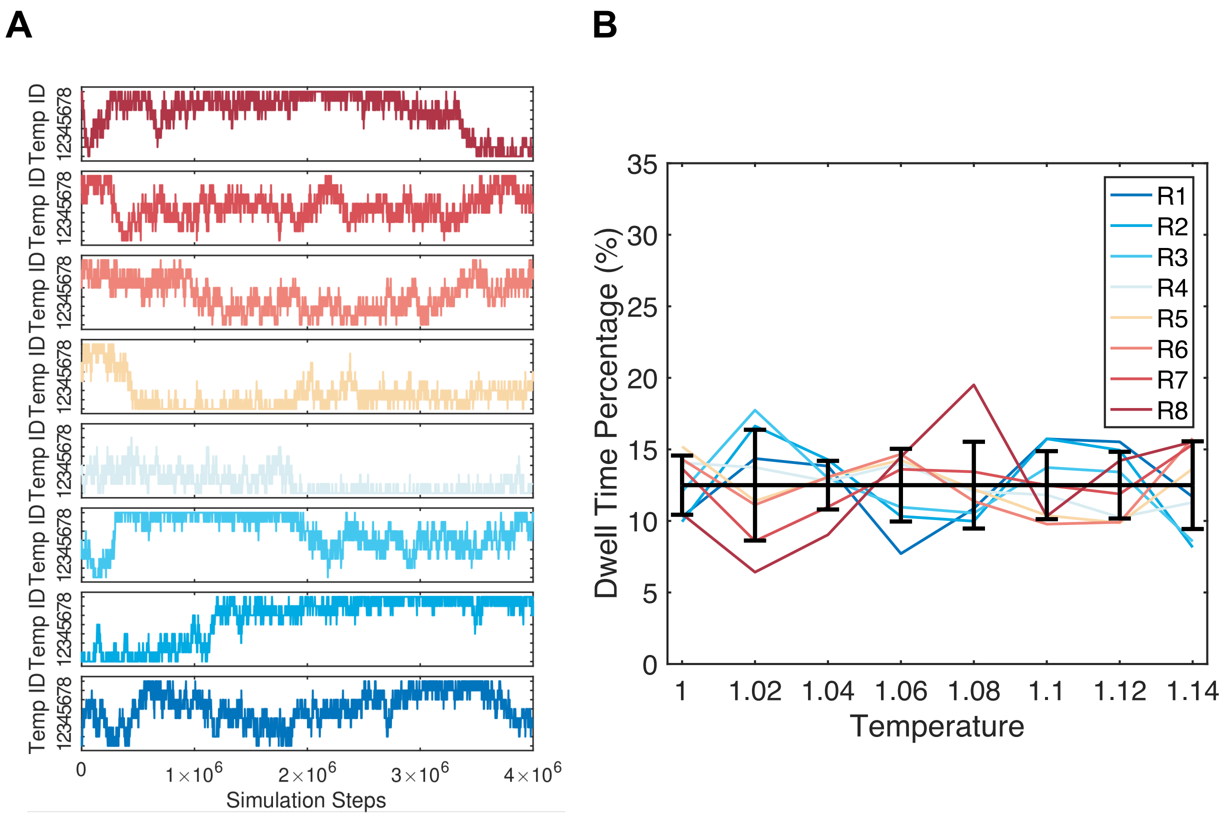

FIG. S21: **Frequent exchanges between replicas were observed during molecular dynamics simulations.** (A) Temperature ID assigned to each one of the eight replicas as a function of simulation time. The temperature varies from 1 to 1.14 with an increment of 0.02 from ID 1 to 8. (B) The average dwell time at various temperatures for each replica. The black line corresponds to the average across replicas with errorbars indicating standard deviations across all trajectories in 16 umbrella windows.

[1] Y. Qi, A. Reyes, S. E. Johnstone, M. J. Aryee, B. E. Bernstein, and B. Zhang, Biophysical Journal **119**, 1905 (2020).

[2] C. M. Caragine, S. C. Haley, and A. Zidovska, Physical review letters **121**, 148101 (2018).

[3] M. Ester, H.-P. Kriegel, J. Sander, X. Xu, *et al.*, in *Kdd*, Vol. 96 (1996) pp. 226–231.

[4] I. Lang, M. Scholz, and R. Peters, The Journal of cell biology **102**, 1183 (1986).

[5] L. Liang, X. Wang, X. Da, T. Chen, and W. R. Chen, Journal of Biomedical Optics **14**, 024013 (2009).

[6] M. Platani, I. Goldberg, A. I. Lamond, and J. R. Swedlow, Nature cell biology **4**, 502 (2002).

[7] Y. Tseng, J. S. Lee, T. P. Kole, I. Jiang, and D. Wirtz, Journal of cell science **117**, 2159 (2004).

[8] N. O. Taylor, M.-T. Wei, H. A. Stone, and C. P. Brangwynne, Biophysical journal **117**, 1285 (2019).

[9] R. Kubo, Reports on progress in physics **29**, 255 (1966).

[10] S. E. Johnstone, A. Reyes, Y. Qi, C. Adriaens, E. Hegazi, K. Pelka, J. H. Chen, L. S. Zou, Y. Drier, V. Hecht, *et al.*, Cell **182**, 1474 (2020).

[11] K. I. Farley, Y. Surovtseva, J. Merkel, and S. J. Baserga, Chromosoma **124**, 323 (2015).

[12] M. Derenzini, L. Montanaro, and D. Trere, Acta histochemica **119**, 190 (2017).

[13] S. E. Weeks, B. J. Metge, and R. S. Samant, Cellular and Molecular Life Sciences **76**, 4511 (2019).

[14] F. Serra, D. Baù, M. Goodstadt, D. Castillo, G. J. Filion, and M. A. Marti-Renom, PLoS computational biology **13**, e1005665 (2017).

[15] T. W. Lion and R. J. Allen, J. Phys. Condens. Matter **24**, 284133 (2012), arXiv:1111.2705.

[16] Y. Zhang, D. S. W. Lee, Y. Meir, C. P. Brangwynne, and N. S. Wingreen, Phys. Rev. Lett. **126**, 258102 (2021).
